# Tree size distribution as the stationary limit of an evolutionary master equation

**DOI:** 10.1038/s41598-024-51553-2

**Published:** 2024-01-12

**Authors:** Szabolcs Kelemen, Máté Józsa, Tibor Hartel, György Csóka, Zoltán Néda

**Affiliations:** 1 Faculty of Physics, Babeş-Bolyai University, Cluj-Napoca, 400347 Romania; 2 Faculty of Environmental Science and Engineering, Babeş-Bolyai University, Cluj-Napoca, 400294 Romania; 3 Forest Research Institute, University of Sopron, Mátrafüred, Sopron, 3232 Hungary

**Keywords:** Population dynamics, Dynamical systems, Statistics, Statistical physics, thermodynamics and nonlinear dynamics

## Abstract

The diameter distribution of a given species of deciduous trees is well approximated by a Gamma distribution. Here we give new experimental evidence for this conjecture by analyzing deciduous tree size data in mature semi-natural forest and ancient, traditionally managed wood-pasture from Central Europe. These distribution functions collapse on a universal shape if the tree sizes are normalized to the mean value in the considered sample. A new evolutionary master equation is used to model the observed distribution. The model incorporates four ecological processes: tree growth, mortality, recruitment, and diversification. Utilizing simple and realistic kernel functions describing the first three, along with an assumed multiplicative dilution due to diversification, the stationary solution of the master equation yields the experimentally observed Gamma distribution. The model as it is formulated allows an analytically compact solution and has only two fitting parameters whose values are consistent with the experimental data related to these processes. We found that the equilibrium size distribution of tree species with different ecology, originating from two contrastingly different semi-natural ecosystem types can be accurately described by a single dynamical mean-field model.

## Introduction

The concept of universality in biological and social systems is highly debated^1–7^. Although many areas of science are keen to uncover universal statistical features of their studied systems, biology, and sociology are usually focusing on quite the opposite, i.e. the contextual specificities of the investigated problem. Besides this dominating trend, in ecology, there are many attempts for a unified statistical description of large plant or animal ensembles. Examples are population abundance studies^8–12^, scaling laws for size^13^, life expectancy or motion trajectories^4,6^, topological features of food and metabolic networks, and emerging patterns. In such a line of studies tree size evolution and the resulting statistics have been intensively studied in the past decades^14,15^. Most of the models used in the literature are motivated by applications in sustainable forest management plans^16^ , or by tree demographic studies^17^.

Tree growth and mortality play a fundamental role in the ecosystem identity as well as the dynamics of forests and woodlands^18^. Exploring the potential universality of the dynamical mechanisms of tree ensembles (compact tree stocks) with different management and natural histories, but belonging to the same bioclimatic region, through simple variables such as the tree size remains an important statistical and modeling challenge^19^. Besides this, there is a general trend in focusing on forest ecosystems, while it is known that trees can take important role in the identity of the open landscapes (see e.g. wood-pasture systems of Europe^20^). With this study we aim to address tree size distribution in two ecologically contrasting ecosystem types from Eastern Europe: semi-natural forests with mature trees and ancient wood-pastures. We selected tree species with different ecological recruitments but that occurs in both ecosystem types. By validating the models and their assumptions on such statistical data one can then step further with the models and study the response of the system to environmental changes and human influence. Assuming argumentable growth, mortality and recruitment rates, here we consider an analytically solvable evolutionary equation to model tree-size statistics in temperate zone woodlands.

**Figure 1:**
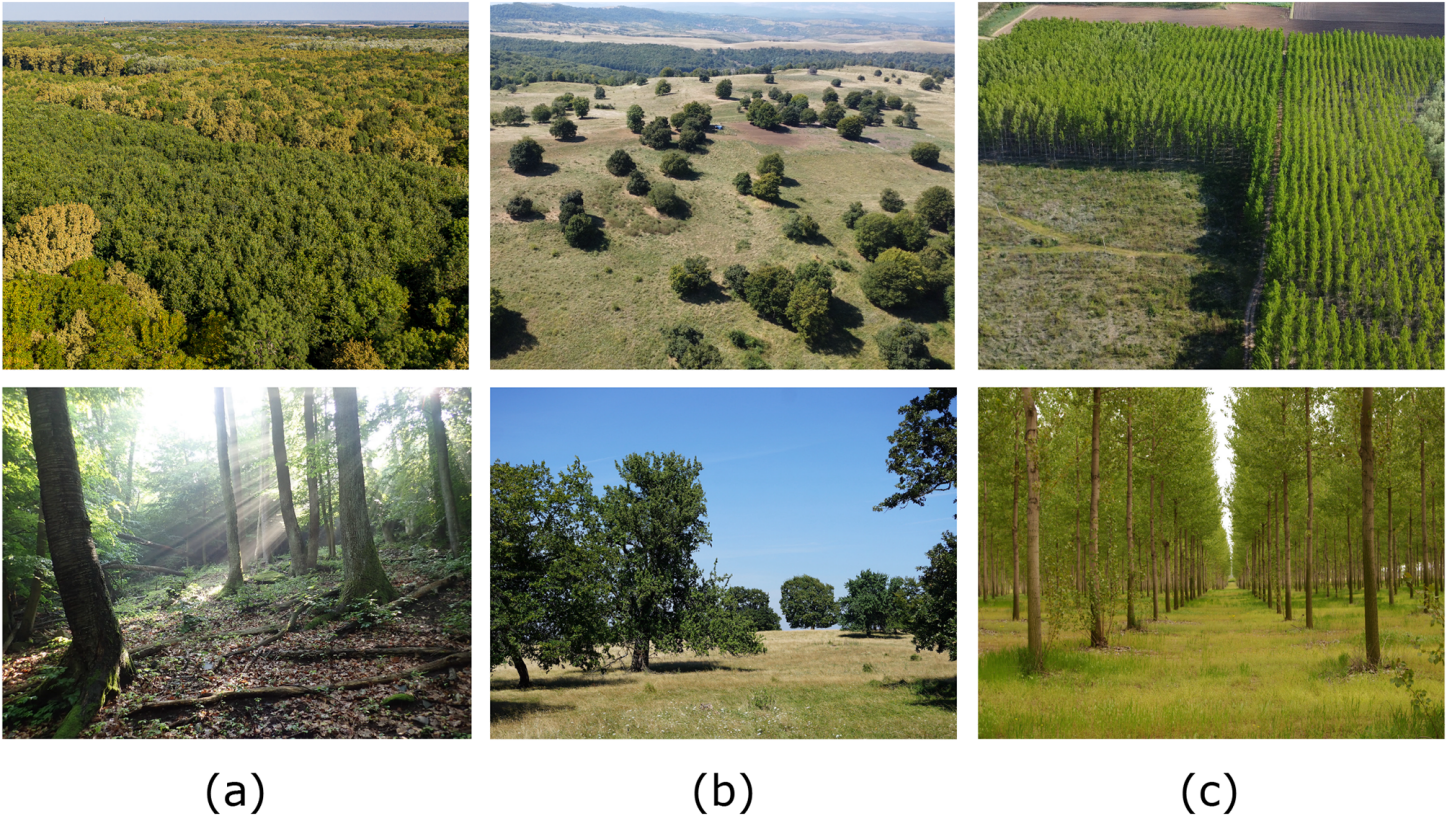
Aerial (upper row) and ground level (bottom row) perspective image of the three ecosystems: semi-natural forest (**a**), semi-natural wood-pasture (**b**), plantation (**c**). Source: Authors.

Earlier statistical studies revealed that a Gamma distribution describes well tree diameter distribution in deciduous forests, although many other fitting functions were proposed^15,21,22^. A particular example of an alternative result is the Weibull distribution applied to DBH distribution of deciduous forests in North America^23^. Building on this finding, we employ a newly developed Local Growth and Global Reset (LGGR)^24^ model which is a simple evolutionary master equation with realistic dynamical assumptions^24,25^ to test the region-specific generality of tree diameter distribution originating from closed canopy mature semi-natural forests and ancient wood-pastures from the continental biogeographic region of Central Europe. Our data on individual tree diameters originates from temperate deciduous forests and wood-pastures covering a complete gradient of management history, from plantation forests (full human control), through semi-natural forests (reduced human interventions, multi-century continuity) to ancient wood-pastures with large old trees (Fig. 1). In the following, first, we provide a description of the study sites, the particularities of the systems, and the origin of the tree size data and then we will apply our model to analytically approximate the observed distributions and the real-life processes that are incorporated in the model.

## Materials and methods

### Tree-size distribution revealed by the experiments

Three different temperate zone woodland ecosystem types were selected for the tree-size measurements, with the aim of mapping various contributions to tree growth, mortality and recruitment processes. We determined the mean Diameter at Breast Height (DBH) for all trees in compact, well-delimited regions for all the studied ecosystems.

Below we describe the three studied systems while the descriptive statistics of the trees are presented in Table 1. The first sample of trees originates from semi-natural, mature, deciduous forest plots (hereafter ,,forest”) from Central Romania (cca 400–600 m asl, Fig. 1a). The dominant native tree taxa that provides identity for these forests are the Oak (*Quercus* sp., hereafter *Quercus*), the Hornbeam (*Carpinus* sp., hereafter *Carpinus*), and the Beech (*Fagus* sp., hereafter *Fagus*). From the perspective of the management history of these forests, only *Quercus* was planted by the Transylvanian Saxons, the other two species were naturally regenerated (intentionally in the case of*Fagus* and unintentionally in the case of *Carpinus*). The natural values of these forests are exceptionally high due to the low human interventions in the past century which allowed the accumulation of dead wood and also the presence of large old trees^26^. Forests from this region are covered by Natura 2000 protected area regulations. Grazing has been prohibited in these forests since cca one century while the main economic use of the trees is the timber^27^. The density of trees is typically higher than 600 trees/hectare^26^. The circumference of trees having at least 3 m height was measured at 130 cm from the ground^28^. Trees from 15 forest plots were measured. Only the measurements from the dominant tree taxa (see above) were used in this study in order to ensure an adequate sample size.

In order to avoid the forest edge effects on tree size the tree measurement plots were situated at a distance of 270–850 m from the forest edge^28^. Based on the in situ age estimation on ring counts, the trees in our sample had between 15 and 250 years. Other, naturally established tree species that could present competition for the modeled trees are: *Acer pseudoplatanus, Acer platanoides, Tilia cordata* and in lesser extent *Prunus avium, Fraxinus excelsior* and *Acer campestre*.

The second sample of trees originates from an ancient, traditionally managed wood-pasture (hereafter wood-pasture) from Central Romania (cca 400–600 m asl, Fig. 1b). The dominant *native* tree taxa in the wood-pasture systems contains the three taxa mentioned above (*Quercus, Fagus, Carpinus*), and measurements of trees belonging to these taxa were used in this analysis. The origin of these wood-pastures is the centuries-long silvopastoral use, when trees regenerated naturally, facilitated by thorny shrubs and periodical reduction of grazing pressure. Similarly to forests, the wood-pastures from this region are covered by Natura 2000 regulations. Unlike in the case of the forests (see above), the main use of trees historically and now is the shade for livestock, fruits, and erosion control for the soil^27^. The density of trees is much below that of forests, being around 7-25 trees/hectare^28^. The circumference of trees having at least 3 m height was measured at 130 cm from the ground^28^ in 40 plots. The age of the trees based on ring counts ranges between cca 10 years to up to 300 years. Other, naturally established tree species that are commonly accompanying the above trees are *Acer campestre, Pyrus pyraster, P. communis, Malus sylvestris, Prunus avium*^28^. For simplicity, whenever we refer to the forest and wood-pasture systems together, we use the term *woodland* in the following.

Finally, in order to have a sharply contrasting system for comparison, we considered monocultures of hybrid Poplar tree (hereafter *Populus*) plantations with a density of approx. 400 trees/hectare, where all trees were planted in the same year and where no human intervention was considered since. The latter measurements aimed to illustrate disparities in tree size distribution within controlled ecosystems that had not achieved statistical stationarity, as opposed to mature natural forest environments characterized by uncontrolled tree diversity and growth, where it is presumed that the tree-size distribution is in a stationary state. Another reason for studying such systems was to have information on the growth dynamics of genetically identical trees in controlled environments. The trees were planted in a regular square grid with an approximate distance of 5 m between each other as it is illustrated in an aerial perspective in Fig. 1c. We made measurements for two plantations of different ages (approximately 10 and 15 years). Since virtually no other tree species were present in the plantations, we assume no interspecific competition in this system.

**Table 1:** Statistical overview of the processed semi-natural woodland and plantation data.

Woodland type	Species/stand age	Nr. of trees	Lowest DBH [cm]	Greatest DBH [cm]	$\langle$ DBH $\rangle$ [cm]
Semi-natural forest	*Quercus*	883	3.2	122.5	38.1
	*Fagus*	1782	3.2	115.2	31.5
	*Carpinus*	1994	1.6	76.4	20.1
Wood-pasture	*Quercus*	1013	4.1	248.3	87.0
	*Fagus*	100	10.2	136.9	74.1
	*Carpinus*	255	4.8	202.1	54.0
Poplar plantation	$\simeq 10 years$	1076	5.7	36.0	18.3
	$\simeq 15 years$	1613	5.1	54.7	27.5

**Figure 2:**
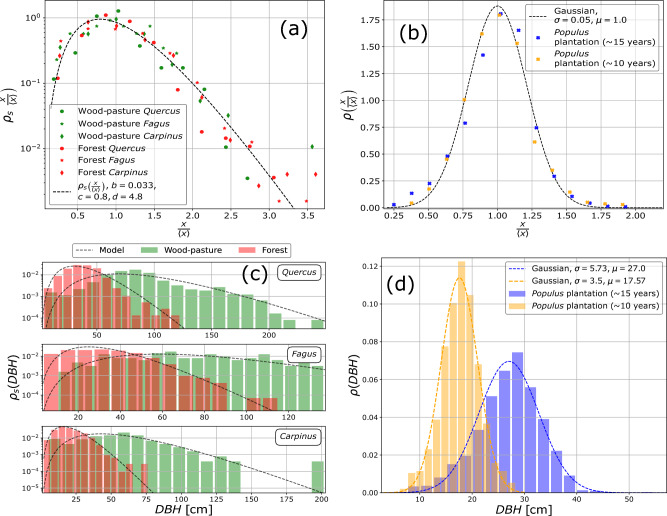
DBH distributions, represented as probability density functions, derived from experimental data. In the first column (**a**, **c**), the distributions for natural forests (in red) and wood-pastures (in green) are depicted alongside the Gamma fit obtained from the LGGR model (Equation 25). The second column (**b**, **d**) shows the DBH distribution within a 10- (in yellow) and a 15-year-old (in blue) *Populus* tree plantation, both with very similar ecological backgrounds, fitted with Gaussian distributions. The lower panels (**c**, **d**) provide an additional visual representation highlighting differences in mean DBH values and illustrating the presence of empty bins.

All three databases constructed by us contain exhaustive measurements in a compact tree ensemble for DBH values^29^. From the collected data we constructed the normalized probability density function for the tree size distribution. Tree sizes, *x*, are quantified with their DBH values, and in our statistics, these were normalized to the mean for the specific tree ensemble: $x \rightarrow y=\frac{x}{<x>}$. The $\rho (y)$ probability densities computed from the data are shown in Fig. 2. The tree size distributions for semi-natural forests and wood-pastures collapse on a master trend which can be well approximated with a Gamma distribution. Our finding on the goodness of the Gamma distribution is in agreement with earlier studies on tree-size distribution in forest environment^15,22^. As expected, the statistics for the plantation is strikingly different, resembling a Gaussian trend (Fig. 2b–d), and the distributions in *y* for two different aged poplar plantations collapse again (Fig. 2b). The Gaussian nature of the distribution in the plantation seems consistent with what one would expect from simple analogies with similar statistics in other controlled biological systems^30^. The Gamma-type tree-size distribution in the forest is however a more complex problem, and in understanding it one should follow the dynamical evolution of the tree ensemble, the interplay of growth and mortality processes. Due to the mature nature of the forest and wood-pasture, one can then assume that the observed distributions are stationary ones, so the stationary limit of such an evolutionary equation should describe the observed distributions, which is a helpful assumption for modeling purposes. In the following we will look deeper into the available statistical data on such systems and try to understand them through mean-field-like evolutionary models.

### The LGGR modeling framework

For modeling purposes we used the Local Growth and Global Reset (LGGR) master-equation framework. This evolutionary type equation is a mean-field-like description of an ensemble where individuals are subject to the same probabilistic local growth and global reset processes^24,31^. Reset is a process where an individual with a given state leaves the considered ensemble (either by mortality or some equivalent process) and it is replaced by a different individual in the ground state. For a unidirectional growth process, this reset is needed in order, to achieve a stationary state. It has been proven to be appropriate for explaining various distributions that are characteristic of different complex systems^24,32,33^. For illustrating such a dynamics let us consider that the states of the elements are characterized by a quantity *x*, in our case this quantity can be the size quantified by DBH.

In a first approach let us discretize the trees’ diameter in well-distinguishable states, described by an integer number of corresponding DBH quanta, *n* ($x\rightarrow n$). In this discrete scenario we denote by $N_n(t)$ the number of elements in state *n* at time *t*. Assuming local probabilistic changes for the states of the elements and a probabilistic resetting process to the $n=0$ state, an evolutionary master equation can be considered:

1 $$\begin{aligned} \frac{dN_n(t)}{dt}=\mu _{n-1} N_{n-1}+\lambda _{n+1}N_{n+1}-(\mu _n+\lambda _n+\gamma _n)N_n(t)+ N_{total} \delta _{n,0}\langle \gamma \rangle (t). \end{aligned}$$

Here $\mu _n$ is the state-dependent local growth rate (probability per unit time) of going from state *n* to state $n+1$, $\lambda _n$ is the local decrease rate of going from state *n* to state $n-1$, and $\gamma _n$ is the reset rate for going from state *n* to state 0. The system preserves the $N_{total}=\sum _{i} N_i$ elements in the system by the last term, which is nonzero for $n=0$ ($\delta _{n,0}$ being the Kronecker delta symbol). We have thus:

2 $$\begin{aligned} \langle \gamma \rangle (t) =\sum _{j} \gamma _j \frac{N_j(t)}{N_{total}}. \end{aligned}$$

For many real-world processes, like the case of trees, the local dynamics is unidirectional. The living tree’s diameter can only increase, with state-dependent growth rates. This means that in Eq. (1) $\lambda _n=0$ for all *n* states and the process becomes the one we named Local Growth and Global Reset (LGGR) dynamics:

3 $$\begin{aligned} \frac{dN_n(t)}{dt}=\mu _{n-1} N_{n-1}-(\mu _n+\gamma _n)N_n(t)+ N_{total} \delta _{n,0}\langle \gamma \rangle (t). \end{aligned}$$

We can switch now the description from the $N_n$ occupancy numbers to the $P_n=N_n/N_{total}$ probabilities that a tree’s DBH is *n* quanta at time moment *t*. Naturally, normalization of $P_n(t)$ satisfies: $\sum _{\{n\}} P_n(t)=1$. The evolutionary master equation describing the local unidirectional transitions and a random resetting process is also a system of coupled first-order differential equations:

4 $$\begin{aligned} \frac{dP_n(t)}{dt}=\mu _{n-1}P_{n-1}(t)-\mu _n P_n(t)-\gamma _nP_n(t) + \delta _{n,0}\langle \gamma \rangle (t) . \end{aligned}$$

The last term in Eq. (4) maintaining the normalization of $P_n(t)$ is :

5 $$\begin{aligned} \langle \gamma \rangle (t) =\sum _{j} \gamma _j P_j(t). \end{aligned}$$

**Figure 3:**
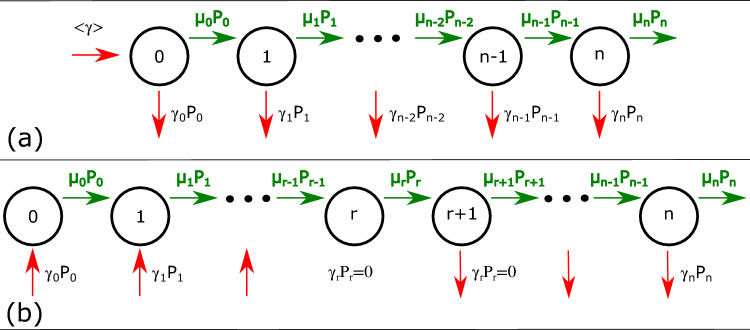
Schematic illustration of the growth and reset process for two scenarios based on the form of the reset rate: (**a**) simple mechanism with only positive reset rate, (**b**) the reset rate can be both negative and positive ($\gamma _n<0$ if $n<n_r$, and $\gamma _n>0$ for $n>n_r$).

Based on the mathematical form of the reset rate, $\gamma _n$, two different dynamical scenarios can be distinguished. The simplest case is when for all *n* values the state-dependent reset rate, $\gamma _n$, is positive. Reset means that the element disappears from state *n* and reapers in state 0. For trees this simple reset describes tree mortality, and consequently the replacement of a tree with a new individual with 0 size. This dynamics is represented in Fig. 3a. A more complicated dynamical scenario is when the reset rate, $\gamma _n$, can be both positive and negative as a function of the *n* value. A scenario of this type is represented in Fig. 3b. One should keep in mind that a negative reset is an inverse process to the ordinary reset, it means that an element is appearing in state *n* and disappears from another state, preserving the total balance. In the case of tree ecosystems this would mean that a new tree that appears in our statistics is characterized not by a 0 size, but it appears in the $n>0$ bin, usually *n* smaller than a critical $n_r$ value. Simultaneously, large trees are dying out or get harvested so they disappear from states with $n>n_r$. This second scenario considering a state-dependent smart reset rate offers much more flexibility and it is more appropriate for modeling the tree growth dynamics in the ecosystems where our data was collected from. Such an attempt was considered recently for modeling the distribution of wealth and income in human societies^33,34^.

Another possibility to include additional terms in the evolutionary equation is by considering the case when the number of elements is also changing in the system. For example, in the case when the number of elements (trees) is increasing (or decreasing) multiplicatively

6 $$\begin{aligned} \frac{dN_{total}}{dt}=\kappa _0 N_{total}(t), \end{aligned}$$

one gets

7 $$\begin{aligned} \frac{dN_n(t)}{dt}=\frac{d\, (N_{total}(t) P_n(t))}{dt}=N_{total}(t)\frac{dP_n(t)}{dt}+P_n(t)\frac{dN_{total}(t)}{dt}=N_{total}(t)\frac{dP_n(t)}{dt}+\kappa _0 N_{total}(t){P_n(t)}, \end{aligned}$$

leading to an extra reset-type term in the master equation for $P_n(t)$:

8 $$\begin{aligned} \frac{dP_n(t)}{dt}=\mu _{n-1}P_{n-1}(t)-\mu _n P_n(t)-(\gamma _n+\kappa _0)P_n(t) + \delta _{n,0}\langle \gamma \rangle (t). \end{aligned}$$

Such kind of process was recently considered for explaining the universal statistics of citations and Facebook shares^35^.

Handling mathematically the coupled differential equations from Eq. (8) in the discrete dynamical picture is quite tedious. The discrete process described by Eq. (8) can be generalized to continuous states ($n \rightarrow x$) in the limit $dt \rightarrow 0$^25^. In such a picture, instead of the discrete state probabilities $P_n(t)$ we will have the continuous probability densities $\rho (x,t)$ with the normalization condition $\int _{\{x\}} \rho (x,t) dx=1$. The growth and reset rates are written as functions of the state variable *x*:

9 $$\begin{aligned} \begin{aligned} \mu _{n}&\rightarrow \mu (x) \\ \gamma _n&\rightarrow \gamma (x) \\ \kappa _0&\rightarrow \kappa . \end{aligned} \end{aligned}$$

By taking this continuous state generalization, the master equation written in Eq. (4) transforms into a partial differential equation:

10 $$\begin{aligned} \frac{\partial \rho (x,t)}{\partial t}=-\frac{\partial }{\partial x} \left[ \mu (x) \rho (x,t) \right] - (\gamma (x)+\kappa ) \rho (x,t) +\langle \gamma (x) \rangle (t) \delta (x). \end{aligned}$$

In this continuous limit, the last term is again the feeding at $x=0$ imposed by the Dirac delta function $\delta (x)$. This term allows to preserve the normalization of $\rho (x,t)$. The mean value of the reset rate ($\langle \gamma \rangle$) is given as:

11 $$\begin{aligned} \langle \gamma (x) \rangle (t) = \int _{\{x\}} (\gamma (x)+\kappa ) \rho (x,t) dx \end{aligned}$$

In the stationary limit

12 $$\begin{aligned} \frac{\partial \rho (x,t)}{\partial t}=0, \end{aligned}$$

the evolution equation for the probability density of having a tree size $x>0$ described by Eq. (10) simplifies into:

13 $$\begin{aligned} \frac{\partial }{\partial x} \left[ \mu (x) \rho (x,t) \right] =- (\gamma (x)+\kappa ) \rho (x,t)). \end{aligned}$$

Equation (13) has a compact analytical solution that depends only on the form of the chosen growth and reset rates^24,25,31^:

14 $$\begin{aligned} \rho _s(x) \, = \, \frac{C}{\mu (x)} \, \textrm{e}^{-\int _{\{x\}}\frac{(\gamma (u)+\kappa )}{\mu (u)}du} , \end{aligned}$$

with *C* being a normalization constant. This closely resembles the formulation proposed by Van Sickle et al.^36^ widely used in demographic modeling for biological systems^37,38^.

Based on the form of the $\mu (x)$ growth- and $\gamma (x)$ reset rates, the LGGR model is able to reproduce stationary probability distributions, $\rho _s(x)$, that are frequently encountered in complex systems^24,25,32,33^. The LGGR’s mathematical apparatus has been comprehensively studied in recent years^24,25,31,39,40^, encompassing aspects of convergence and applicability to various fields of science^24,32–35,41^.

### Compliance statement

Our research, involving non-invasive measurements, fully adheres to the regulations of the International Union for Conservation of Nature (IUCN) Policy on Species at Risk of Extinction and the Convention on the Trade in Endangered Species of Wild Fauna and Flora (CITES) to ensure the ethical treatment and protection of endangered plant species.

## Results

We apply now the LGGR modeling framework to describe the dynamics of tree-size distribution. There are three main processes that drive this dynamics: a monotonic growth, the possibility of a reset (natural mortality or exploitation followed by the recruitment of new trees), and a multiplicative change in the number of trees belonging to one species. These stochastic processes are mathematically quantified by the $\mu (x)$ growth rate, the $\gamma (x)$ reset rate and $\kappa$ dilution rate. Once the needed kernel functions are realistically defined, the dynamics given by the LGGR model should yield the time evolution of the tree-size distribution function. In a general study of the LGGR dynamics it was previously shown^40^, that apart from some pathologic cases, such systems are indeed converging to the stationary distribution. Depending on the starting condition, the mean of the distribution might converge slowly to a stationary value, however, the distribution of $x/\langle x \rangle$ converges quickly to a stationary distribution. Given that the considered ecosystems (forest and wood-pasture) are determined largely by mature trees, we can assume that the DBH distributions that we see in the forest and wood-pasture correspond to the stationary distribution. This is different for even-aged plantations, which are still in continuous development. Interestingly however, even in this clearly non-stationary case, their size distribution during the growth process can be rescaled if we normalize the sizes to the mean value. This is what we see in Fig. 2b for the plantations: although the diameters are continuously increasing, the statistics in $x/\langle x \rangle$ is only slightly different for a plantation that is 10 or 15 years old. This scaling, suggests that the growth speed of the trees has to increase as a function of the tree diameter, i.e. larger trees have to grow faster.

For choosing the right functional form for the growth and reset rates we take into account empirical knowledge of the tree life cycle, diversity dynamics in natural forest environments, previous experimental observations on such processes, and aim for a mathematical simplicity that allows compact analytical results. We follow here a physicist approach for such complex systems, using a small number of model parameters, and by simple, yet realistic, assumptions we aim to describe the main elements and universal features in the observed statistics. The confirmation of our model will not focus thus on the statistical goodness of the fit as it was done in the work of Lima^21^ for example, but rather on the desire to understand by a simple analytical model the dynamical mechanism leading to the universal form of the tree-size distribution in the studied forest and wood-pasture ecosystems.

### Growth rate

Both our measurement data on the Populus plantations (demonstrating an increasing standard deviation with mean size increment; see Fig. 2d) and the data available in the literature^11,13,42–49^ supports the assumption that the growth rate ($\mu (x)$) of deciduous trees monotonically increases with the tree diameter. Even without a reset process, this increase cannot go on indefinitely, therefore for large trees, it has to saturate. A mathematical form that can accommodate such a growth rate is:

15 $$\begin{aligned} \mu (x) = d_{1}\frac{x}{x+b}, \quad b \ge 0 \end{aligned}$$

**Figure 4:**
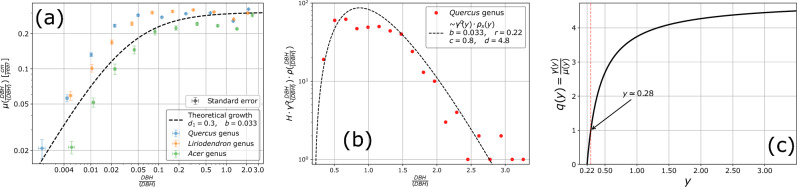
Supporting data and illustrations for the modeling assumptions: (**a**) Growth rate determined from the width of tree rings. The panel illustrates on log-log scale the width of tree rings as a function of stem diameter at one meter above the ground for three tree genera as indicated in the legend. The trend illustrated by the dashed line is given by Eq. (15) with parameters indicated in the figure. The error bars around the data points illustrate the standard error. (**b**) Consistency between the dead trees size distribution, the considered reset rate, and fitted tree-size distribution. Histogram of the size distribution (sizes normalized to the mean) of dead Quercus trees from censused forests plotted together with the fit given by $H \cdot \rho _s(y) \gamma '(y)$ with $r=0.22$ and parameters estimated from the probability density function. *H* is a proportionality constant needed to fit the experimental histogram. The data used for figures in panels (**a**) and (**b**) was provided by^50,51^. (**c**) Reset over growth rates probability for the genus Quercus as a function of the trees $y=x/\langle x \rangle$ relative size. The trend of $q(y)=\gamma '(y)/\mu (y)=d(y-r)/y$ for $r=0.22$ and $d=4.8$.

The specific functional form, Eq. (15, for the growth rate was taken by aiming to mathematical simplicity. However, its form and the involved *b* parameter value are consistent with all experimental data (supporting information also for a similar sub-linear growth rate from Moore et al.^11^). The growth rate given in Eq. (15) is supported by the data provided by the United States National Park Service (NPS)^50,51^, where we have identified the annual growth rate from the diameter of the tree rings. For three tree genera (*Quercus sp., Liriodendron sp., and Acer sp.*) in Fig. 4a we plot the averaged annual growth rate as a function of $DBH/\langle DBH\rangle$ (*DBH* measured here approximately 1 m above the ground). The numbers of trees by genera that were considered for computing these growth rates were: for *Quercus genus* 545 trees (*(Quercus alba, Quercus rubra, Quercus montana* species); for *Liriodendron genus* 210 trees (*Liriodendron tulipifera species*); for *Acer genus* 64 trees (*Acer negundo, Acer rubrum, Acer saccharinum* species). In Fig. 4a, we also indicate the trend that is given by the kernel function for the growth rate, Eq. (15), with a parameter set that gives a reasonable description of the data.

### Reset rate

Unlike the growth rate, the reset rate is much more difficult to measure experimentally. In the LGGR framework the reset rate, characterizing the transitions from large to small DBH categories, incorporates the combination of the mortality (caused either by natural mortality or forest exploitation) and the recruitment (appearance of young and small trees) processes. This corresponds to the replacement of dead trees with new ones. To realistically choose the form of the reset kernel function ($\gamma (x)$), one should consider both, the form of the mortality rate and the recruitment rate in the function of tree size. The recruitment rate acts as a negative reset rate in this context. Similarly with the increasing growth rate as a function of tree sizes, assuming an increasing reset rate would be natural. One would expect that the reset rate is also converging to a constant value for very large trees. Deriving a reasonable kernel function for the reset rate can adhere to the following logic:

*First*, in all tree census data there is an $x_{min}$ minimal diameter under which trees do not enter in the statistics both for the dead and living trees. This means that from the viewpoint of the detected dynamics the reset should be negative (trees are just entering in the statistics) for $x<x_{min}$. The recruitment of new trees happens in this experimentally less tracked DBH region. Thus, the available data does not reflect the reset rate ($\gamma (x)$) itself, it yields instead the probability that a dead tree with a given diameter exists in an ecosystem. Therefore, this probability is rather related to the mortality component of the reset rate^52–55^. In the framework of our modeling, this quantity is proportional to the product of the reset rate and probability density function, $~ \gamma '(x) \cdot \rho (x)$. As the recruitment rate presents a substantial negative reset for small tree sizes ($x < x_{\text {min}}$) and the mortality rate exhibits a declining pattern^52,53^ for $x > x_{\text {min}}$, the cumulative impact of these functions can indeed be approximated as an increasing but converging function. These facts are all in agreement with an increasing reset rate in the form:

16 $$\begin{aligned} \gamma '(x)=f_1 \frac{x-r}{x+g} \quad r \ge 0; \quad g>0 \end{aligned}$$

Here *r*, *g* and $f_1$, are positive constants. In further calculations we will assume $g=b$, reducing the number of model parameters and making the mathematics simpler.

*Second*, it is known that tree diversity increases with time in both forests and wood-pastures. In closed canopy mature forests key drivers for the establishment of new tree species are the intermediate-level disturbances (typically affecting both the tree stands and the individual mature trees, creating gaps of various sizes), which results in a diversification of the biotic (e.g. herbaceous plants) and abiotic (light, microclimate) conditions at local and plot levels^56^, in the benefit of both shade tolerant and light-demanding trees^57^. In wood-pasture systems, light is rarely a limiting factor for tree establishment. Here the species diversification in time depends on the herbivore density and dynamics as well as the existence of protecting structures for individual trees across the grazed land (e.g. associational resistance assured by unpalatable plants)^58^. Besides the local factors, the natural establishment of new tree species in the two ecosystem types depends on the regional species pool (referred to as ‘external memory’ in^59^). The existing diversification means, that whenever a tree is dying, its place can be overtaken by an individual from another species. In the case of *Quercus* trees, for example, the establishment of young individuals to replace the mature *Quercus* trees in forests is hampered by improper light conditions. In such systems the likelihood for other, shade-tolerant trees to replace the *Quercus* is high. In the case of *Fagus* and *Carpinus*, both species tolerate and regenerate in shade - in these cases, the replacement of old individuals can happen by the same or different species. Additionally, the removal of mature trees represents a diversification of the forest stand for the semi-natural forest. Since the diversity is increasing, this effect will lead to a multiplicative decrease in the number of individuals for a species, which is equivalent (as we have shown in the previous section) with a $\kappa <0$ state independent reset term. Taking all these effects into account, we propose that the reset rate should be taken in the form,

17 $$\begin{aligned} \gamma (x) = \gamma '(x) + \kappa \equiv d_2\frac{x-c}{x+b}, \end{aligned}$$

with:

18 $$\begin{aligned} c= & {} \frac{f_1r-b\kappa }{f_1+\kappa }> r >0, \end{aligned}$$

19 $$\begin{aligned} d_2= & {} (f_1+\kappa )<f_1; \quad \text {and} \quad d_2 >0. \end{aligned}$$

Using data for the size distribution of dead trees in several mature deciduous forests we can also verify whether the form of the proposed reset rate is a reasonable hypothesis. If we denote by $\rho _s(x)$ the stationary limit of the probability density for the DBH of the trees, the size distribution of the dead trees should follow the $\rho _s(x) \cdot \gamma '(x)$ distribution with $\gamma '(x)$ given by Eq. (16). For testing this reset rate we can use again the data from NPS^50,51^ for dead trees diameter, which should be fitted as $\rho _s(y) \cdot \gamma '(y)$. The data provided by the United States National Park Service contains the diameter of dead trees within a number of 320 plots from 10 national parks in the USA. For consistency, and for putting together several data from different forests, the trees’ diameter is normalized to the mean value of tree diameters in the forest (taking now only the living trees). Considering the *Quercus* genus, the data for the histogram of the dead trees is plotted in Fig. 4b. The dashed line indicates a fit based on Eq. (25) with the parameters $c=0.8$, $b=0.033$, and $d=4.8$ for the experimentally observed probability density and $r=0.22$ in the $\gamma '(y)$ reset rate.

Concerning the three investigated ecosystems with the applied cultivation approaches, the main triggering conditions for the tree mortality (reset) are summarized in Table 2.

**Table 2:** Type of management control and main causes of tree mortality in the considered woodland areas.

System	Management control	Main drivers of tree mortality (interpretable as a contribution to reset in our modeling)
Mature forest with high natural values	Weak, reduced to an initial *Quercus* plantation in the first part of the 1900s. The subsequent increase in the abundance of *Carpinus*, *Fagus*, and other tree species happened naturally. Natural regeneration and the accumulation of dead trees in the forest are accentuated. While timber exploitation happens (the *Quercus* and *Fagus* being valued), this is never at large scale, only at parcels of cca 1-3 hectares and when the trees have cca 90-120 years. Grazing is prohibited by law^28^.	Mostly inter- and intraspecific competition for light. To a lesser extent extreme meteorological conditions, pest outbreaks, fire, and illegal cutting^28^.
Ancient wood-pasture	Weak, represented by traditional grazing with sheep, cattle, buffalo, and other livestock as well as scrub clearance in the central parts of the pasture. Tree regeneration happens in pulses through associative resistance and in pulses after temporary grazing pressure reductions^28^. The oldest trees in such a system have over 300 years.	Mostly extreme weather conditions (strong winds, lightning, and recently increasing drought) weakening or damaging individual mature trees which will be subsequently removed with formal permit. Illegal fires set by shepherds can be also a cause of mortality for old trees. Grazing prohibits tree regeneration in areas without shrubs. In a lesser extent competition and pests or diseases^28^.
Plantation under strong management	In the case of our two plantations no direct human intervention has happened since the establishment.	The intraspecific (or even intraclonal) competition can be significant. As clone origin, the trees are almost identical genetically. Abiotic factors (wind/storms/snow) caused some level of disturbances.

### Stationary size distribution

Once we accept the form given by Eqs. (15) and (17) for the growth and reset rates, respectively, it is straightforward to compute the stationary probability density, $\rho _s(x)$. Since the $\kappa$ value has been now incorporated in the $\gamma (x)$ reset rate (Eq. 17), according to Eq. (14) we get

20 $$\begin{aligned} \rho _s(x) \, = \, \frac{\mu (0) \rho _s(0)}{\mu (x)} \, \textrm{e}^{-\int _{\{x\}} \frac{\gamma (u)}{\mu (u)}du} = C x^{d \,c-1} (x+b)\, \textrm{e}^{-d \,x} , \end{aligned}$$

where $d=d_2/d_1$ and *C* is a normalization constant. If the distribution is defined on the $x \in [0,\infty )$ interval, the normalization constant becomes:

21 $$\begin{aligned} C=\frac{d^{c\, d}}{(b+c)\Gamma [c\,d]}. \end{aligned}$$

The first moment of the distribution (average) is also analytical:

22 $$\begin{aligned} \langle x \rangle =c \left( 1+\frac{1}{(b+c)d} \right) . \end{aligned}$$

We write now the distribution function for the $y=x/\langle x \rangle$ tree-sizes normalized relative to the mean value:

23 $$\begin{aligned} \rho _s(y)=\frac{d^{c\, d}}{(b+c)\Gamma [c\,d]} \langle x \rangle ^{d c} \textrm{e}^{-d \langle x \rangle y} y^{dc-1} (y \langle x \rangle +b). \end{aligned}$$

Assuming that $\langle x \rangle =1$, it results

24 $$\begin{aligned} b=\frac{c}{(1-c)d}-c, \end{aligned}$$

therefore the probability density function will have only two parameters to fit the experimental results for $y=x/\langle x \rangle$:

25 $$\begin{aligned} \rho _s(y)=\frac{d^{c\, d}}{(\frac{c}{(1-c)d})\Gamma [c\,d]} \textrm{e}^{-d y} y^{dc-1} \left( y+\frac{c}{(1-c)d}-c\right) . \end{aligned}$$

As Fig. 2a shows, the probability density for the distribution of $x/\langle x \rangle$ on forests and wood-pastures collapse, and it can be well approximated by the form given in Eq. (25), with parameters $c=0.8$ and $d=4.8$, leading to $b=0.033$.

**Consistency in the model parameters.** To ensure consistency in the model parameters, we simultaneously considered the goodness of fit for both the experimentally observed probability density functions and the data related to growth and reset processes. The optimal fit parameters were established by minimizing the Root Mean Squared Logarithmic Error through iteration across a fine grid within the parameter space. When selecting the parameters, equal weight was given to the fitting of the growth data in Fig. 4a, the data concerning the reset rate in Fig. 4b, and the DBH distribution data in Fig. 2a. We concurrently minimized the Root Mean Squared Logarithmic Error for all three quantities. Doing so, the fit parameters for the experimentally observed probability density function are in agreement with the data that we have on growth and reset processes. The values of the coefficients of determination ($R^2$) for the obtained fittings are listed in Table 3. Also in agreement with our prediction and imposed restrictions, we find that the best *r* parameter value for fitting the reset data satisfies the $r< c$ condition. Because we have no information on when these trees dried out, no direct values of the rates can be estimated and as a consequence, one cannot determine the $f_1$ parameter that would allow estimation of the $\kappa$ parameter as well.

**Table 3:** Coefficient of determination ($R^2$) calculated for the Gamma fit (Eq. 25) of the tree-size distribution in natural woodlands represented in Fig. 2a; the fitting of the experimentally obtained growth rates by Eq. (15) in Fig. 4a; and the fitting of the size distribution of dead trees by $\gamma '(y)\cdot \rho _s(y)$ in Fig. 4b.

Species	DBH distributions		Growth kernel	Reset kernel
	Forest	Wood-pasture		
*Fagus*	0.80	0.47	–	–
*Carpinus*	0.97	0.85	–	–
*Quercus*	0.91	0.80	0.78	0.60
*Liriodendron*	–	–	0.81	–
*Acer*	–	–	0.72	–

Accepting the $r=0.22$ parameter from the fit in Fig. 4b, we can also predict the reset rate over growth rate ratio ($q=\gamma '(y)/\mu (y)$) as a function of tree diameters (all sizes taken relative to the mean value). We get:

26 $$\begin{aligned} q=d\, \frac{y-r}{y} \end{aligned}$$

Using the *d* value obtained through fitting the experimental data, $d=4.8$, and the $r=0.22$ value the *q*(*y*) trend is plotted in Fig. 4c. From this figure we learn, that the ratio *q* is monotonically increasing as a function of tree sizes and for trees over $y>0.28$ the reset process is more probable than growth. This intuitively explains why despite the monotonically increasing growth rate the forest does not get filled up by very large trees.

In the preceding section, we highlighted the necessity of integrating diversification into the model. Equation 25 defines a stationary probability density function that exhibits its peaks around the value represented by the parameter *c*. When $c=0.8$, as established, the density function shows its highest concentration around this value, closely resembling the experimental data (refer to Fig. 2a). Considering the relationship between the parameter *r* (fixed at 0.22) and $\kappa$, if $\kappa$ were 0, indicating the absence of diversification, Eq. (25) would exhibit a peak around 0.22. However, this projection does not align well with the experimental size distributions presented in Fig. 2a. This reasoning underscores the indispensability of incorporating the diversification process into the model.

## Discussion

Tree size diversity patterns in natural deciduous forest and wood-pasture environments is a complex problem, where new data and simple realistic mathematical models are needed for its better understanding. It has been conjectured that the diameter distribution of trees belonging to given deciduous species follows a Gamma distribution in a mature natural forest^15,21,22^. Here we brought new evidence supporting this hypothesis, considering new exhaustive measurement data for three tree taxa in two different environments: mature semi-natural forests and wood-pastures located in central Romania.

Apart from the generality for the Gamma distribution, our data suggests an intriguing statistical universality: rescaling the tree diameters with the average tree diameter for that species in the given ecosystem type, all the data collapsed on the very same distribution. This intriguing universality across these sites is captured by our model if we assume the same *c* and *d* parameters (Eq. 25) for all taxa and for the different environments (forest and wood-pastures). This means that in the tree census one has to consider the same lower limits for recording a tree, the same dilution rate, $\kappa$, due to diversification, and the ratio of the reset and growth rates should be similar for the same $y=x/\langle x \rangle$ relative diameter values. These are all in agreement with the fact that the considered three deciduous genera dominate quite equally these treed environments and they are ecologically equally fit. Seemingly we deal thus with some interesting stylized facts in tree-size diversity patterns for deciduous temperate climate woodlands (forests and wood-pastures), allowing also a useful rescaling among different species and different semi-natural ecosystems. Data collected on relatively young (up to 15 years) tree plantations reveal different size diversity patterns (i.e. Normal distribution). These plantations clearly did not reach maturity and a stationary state, therefore the difference relative to what is observed in the other two environments should not be a surprise at all. These findings suggest that the Gamma type fit for the tree-size distribution can be used as a simple proxy to infer natural or close-to-natural dynamics of tree establishment and growth. Asymmetric competition (characteristic of natural ecosystems) can result in higher tree size inequality (as found by us for the forest and wood-pasture compared to plantations) where the tree regeneration and growth patterns are determined by largely natural interactions between the trees^60^.

In order to understand theoretically the tree-size distribution in semi-natural woodland environments the main processes that govern the evolution of the tree ensemble have to be considered. The first process is a *monotonic growth*, which was assumed to increase with tree size and saturate in the limit of large diameters. For analytical simplicity and in agreement with supporting information from literature^11,42,43,45,46,49^, we chose a simple sub-linear function for the above (Eq. 15). The second and third processes that complement this growth and allow for developing a stationary distribution are *tree mortality* and *recruitment*, captured by our reset rate. In order to derive a mathematical form for this rate, we considered a process where there is a lower *r* limit for detecting a new or dead tree in the census (trees below this size are not measured). According to this methodology below the *r* size, trees appear in the statistics, known as the phenomenon of recruitment, a process that can be taken into account with a negative reset rate. It is assumed that the tree mortality rate should decrease in a woodland environment with tree sizes due to both endogenous and exogenous effects^52–55^. As a combination of these two ecological processes, we assumed that, similarly to the growth rate, the reset rate should increasingly saturate to a constant value for large trees. A mathematically simple reset rate that could reproduce these features was proposed in the form given by Eq. (16). As we have emphasized in the previous section, this reset rate together with the proposed form of the probability density function (Eq. 25) leads to results that are in agreement with observations (Fig. 4b). Finally, in order to explain the large $c=0.8$ value in the final form of the reset rate (Eq. 17), which is necessary for a reasonably good fit of the diameter distributions, we had to assume another reset-like process, due to the *diversification process* implying competitive exclusion of certain species by other species. As it was shown in the general discussion (The LGGR modeling framework), a multiplicative growth or dilution in the total tree number belonging to a species is equivalent to a reset term in the master equation for the probability density function.

The easiest way to elaborate a model that is able to predict a stationary tree-size distribution is to incorporate these probabilistic processes in an evolutionary master equation. This has been done here, in the framework of the previously introduced LGGR model^24^. We considered mathematically simple yet realistic forms for growth ($\mu (x)$) and reset ($\gamma (x)$) rates, as convenient first-order approximations supported also by experimental data. The stationary distribution provided by the LGGR model reproduced successfully the experimental results. Our main interest focused on unveiling some interesting universality and showing the visually acceptable collapse of the renormalized data. In fitting the experimental data and analyzing the goodness of the fit our aims were quite modest and we followed basically a physicist modeling methodology. Instead of a rigorous quantitative modeling with many unknown parameters, we opted for an analytically solvable model with basically two free parameters. Based on the literature, similar elegant approaches have been favored by others as well^11,23^. By doing this we concentrated less on the statistical goodness of the provided fit and insisted more on modeling consistency and the usefulness of analytical results in a compact mathematical form. Definitely, one can come up with other, more accurate forms for these kernel functions, describing better the experimental data. The drawback of such an attempt will be the more complicated form for the stationary probability density and the inevitable increase in the number of model parameters. The available DBH data itself was barely enough to construct the qualitative form of the probability density functions, and as it is visible in Fig. 2a it has large deviations from a smooth trend. Taking into account also that the experimental data used for testing the growth and reset rate is quite poor and their sources are diverse, we consider that this consistent theoretical description is more fruitful for understanding the experimentally observed universal shape of tree-size distributions across these sites.

Naturally, in order to get further confidence in the proposed model, new and good-quality data should still be gathered. It would be interesting to test in the very same forest and wood-pasture environment the growth and reset dynamics of the considered tree species. Within the same woodland ecosystem, it would be also interesting to gather quantitative data on the diversification process for the tree species. To do this, however, controlled tree census measurements have to be planned and continuously repeated.

## Data Availability

The data collected by the authors (summarized in Table 1) are freely available for download from ^29^. The data used for plotting Figs. 4 and 4b are from the mentioned sources, and can be obtained by request.
